# Health care access dimensions and cervical cancer screening in South Africa: analysis of the world health survey

**DOI:** 10.1186/s12889-015-1686-5

**Published:** 2015-04-15

**Authors:** Tomi F Akinyemiju, Jasmine A McDonald, Paula M Lantz

**Affiliations:** Department of Epidemiology, University of Alabama at Birmingham, Birmingham, AL USA; Department of Epidemiology, Columbia University Medical Center; Mailman School of Public Health, New York, NY USA; Department of Health Policy and Management, Milken Institute School of Public Health, the George Washington University, Washington, DC USA

**Keywords:** Cervical cancer screening, Health care access, Sub-Saharan Africa

## Abstract

**Background:**

Cervical cancer is the most commonly diagnosed cancer and the leading cause of cancer mortality among women in sub-Saharan Africa. Recent recommendations for cervical cancer primary prevention highlight HPV vaccination, and secondary prevention through screening. However, few studies have examined the different dimensions of health care access, and how these may influence screening behavior, especially in the context of clinical preventive services.

**Methods:**

Using the 2003 South Africa World Health Survey, we determined the prevalence of cervical cancer screening with pelvic examinations and/or pap smears among women ages 18 years and older. We also examined the association between multiple dimensions of health care access and screening focusing on the affordability, availability, accessibility, accommodation and acceptability components.

**Results:**

About 1 in 4 (25.3%, n = 65) of the women who attended a health care facility in the past year got screened for cervical cancer. Screened women had a significantly higher number of health care providers available compared with unscreened women (mean 125 vs.12, p-value <0.001), and were more likely to have seen a medical doctor compared with nurses/midwives (73.1% vs. 45.9%, p-value = 0.003). In multivariable analysis, every unit increase in the number of health care providers available increased the likelihood of screening by 1% (OR = 1.01, 95% CI: 1.00, 1.01). In addition, seeing a nurse/midwife compared to a medical doctor reduced the likelihood of screening by 87% (OR = 0.13, 95% CI: 0.04, 0.42).

**Conclusions:**

Our findings suggest that cost issues (affordability component) and other patient level factors (captured in the acceptability, accessibility and accommodation components) were less important predictors of screening compared with availability of physicians in this population. Meeting cervical cancer screening and HPV vaccination goals will require significant investments in the health care workforce, improving health care worker density in poor and rural areas, and improved training of the existing workforce.

## Background

Cervical cancer incidence and mortality rates are higher in Africa compared to other parts of the world [1]. Cervical cancer is also the most commonly diagnosed cancer and the leading cause of cancer mortality among women in Africa, with 99,000 new diagnoses and 60,000 deaths in 2012 [2]. Although the number of cases is expected to increase over the next several decades due to population aging, most countries on the continent still lack comprehensive cervical cancer prevention programs [2,3]. Recent recommendations from the World Health Organization International Agency for Research on Cancer (WHO-IARC) include a specific focus on primary prevention through HPV prophylactic vaccination, and secondary prevention through screening in the context of an adequately-resourced health system infrastructure [4,5].

It is well documented that a beleaguered and under-resourced health care infrastructure is a fundamental cause of poor health and high mortality rates in sub-Saharan Africa (SSA) [6-9]. Importantly, the success of any primary or secondary disease prevention effort is intricately linked with the quality and accessibility of health services. However, few studies have comprehensively studied the different dimensions of health care access, and how these may influence health behavior, especially in the context of clinical preventive services [10,11]. Cervical cancer is an ideal case study for examining how different components of health care access influence the utilization of preventive services, as there are well-established and well-accepted screening guidelines. For instance, a recent WHO report indicates that over 80% of all countries had a comprehensive cancer control plan in place [12], in addition to the WHO comprehensive cervical cancer control guide and position papers focusing on cervical cancer prevention guidelines [13,14]. Furthermore, South Africa is an ideal setting for investigating access to health care as a multi-dimensional construct since it is one of the most developed countries in SSA and therefore more likely to have a structured health care system.

In SSA, as well as other regions of the world, access to health care is often misunderstood in discussions about primary and secondary prevention of cancer. Most studies focus on physical and economic access to care as the critical factors in explaining level of and disparities in cancer screening [15-18]. However a more complete and sophisticated approach to the definition of access to care has been missing. According to the Penchansky and Thomas framework (1981,1984), health care access is a latent construct with 5 key dimensions: affordability, availability, accessibility, accommodation and acceptability [19,20]. Affordability refers to the ability and willingness to pay for health service; availability refers to the number and type of health care provider present; accessibility refers to the geographic distance and convenience of travel to the health care center; accommodation refers to the perception of adequacy of health care provider in terms of skills or supplies; and acceptability refers to comfort with health care provider characteristics and perceptions of inferior treatment based on gender, ethnicity or social class. Together these dimensions provide a comprehensive approach to measuring access and understanding how these health care dimensions influence health behaviors while taking into account the cultural, social, economic and physical factors that may influence the perceived benefit of seeking care and the ability to obtain quality care.

Our analysis is focused on secondary prevention (i.e., cervical cancer screening with a pelvic examination and/or a Pap test) of cervical cancer in South Africa. The current WHO recommendation for primary prevention of cervical cancer focuses on the use of HPV vaccination for 9–13 year old girls worldwide, a cost-effective approach that could potentially prevent 70% of all new diagnoses of cervical cancer [13]. However, the full cost of the HPV vaccine ranges between $39 and $300 in high-income countries [21], and unless highly subsidized in low-income countries, cost will be a major barrier to wide availability and adoption [22,23]. Since the HPV vaccine is a relatively new development, it may take some time before it is widely adopted in resource poor areas of SSA, and the success of HPV vaccination as a primary prevention strategy requires deep integration with the existing health care infrastructure. In addition, secondary prevention using pelvic examinations with Pap tests and pelvic examinations with visual inspection and acetic acid (VIA) will continue to be part of a comprehensive cancer control strategy for millions of women who are no longer eligible for HPV vaccination. It is therefore important to examine the association between all dimensions of health care access and cervical cancer screening. These findings will contribute to our understanding of the importance of the health system in the success of future primary or secondary prevention effort for cervical cancer.

## Methods

The South Africa World Health Survey (WHS) was conducted between 2002 and 2004. It was a cross-sectional study of adults ages 18 years and older residing in households selected through a multistage cluster sampling method. Further details of the WHS methodology have been published previously, and is available online [24]. The current analysis was based on de-identified and publicly available WHS data and so was exempt from IRB review, although the initial WHS study was approved by the WHO ethical review board, and informed consent was obtained from each study participant. The WHS data is available for download to researchers after submitting a data request through the World Health Survey website [24]. Study participants were administered a detailed in-person interview that focused on multiple household and individual characteristics, risk factors, and health outcomes.

The outcome of interest in this analysis was self-reported cervical cancer screening based on the receipt of a pelvic examination with or without a Pap test in response to the questions: 1. “When was the last time you had a pelvic examination, if ever? (By pelvic examination, I mean when a doctor or nurse examined your vagina and uterus?).” Only women who reported a pelvic examination within the past 3 years were asked. 2. “The last time you had the pelvic examination, did you have a PAP smear test? (By PAP smear test, I mean did a doctor or nurse use a swab or stick to wipe from inside your vagina, take a sample and send it to a laboratory?).” Cervical cancer screening in this analysis was defined as receipt of a pelvic exam with or without a Pap test in the past 3 years categorized as yes/no.

Socio-demographic characteristics assessed from the questionnaire included marital status (single, married/cohabiting, divorced/separated, widowed), education (primary school or less, secondary school, or college and above), employment (government employee, non-government employee, self-employed, not working for pay), residential region (rural, urban), and overall health (good, moderate, poor). Household socioeconomic status (SES) was defined based on a composite index of permanent household income indicators. This method has been widely used to characterize SES in lower income countries where income and educational level may not adequately capture the full extent of an individual’s socio-economic status [25-27]. Principal components analysis (PCA) was performed on data assessing ownership of assets such as refrigerators, washing machines, mobile phones, chairs, tables, etc. A household SES score was obtained from PCA analysis by weighting each indicator by the coefficient of the first principal component, and each member of the household was assigned the same SES. SES was categorized into quartiles ranging from highest to lowest SES.

All 5 health care access dimensions from the Penchansky and Thomas framework were evaluated from the WHS questionnaire. Questionnaire items regarding the features of the health care system and participant experience were solicited only from women who had been to a health care facility in the past 12 months for their own health or for their children.

All analytic procedures accounted for the complex survey design by using sample weights and stratum codes provided in the dataset, using SAS (version 9.4, SAS Institute, Cary NC). Descriptive statistics were conducted to examine differences between screened and unscreened women by socio-demographic and health care access variables. Multivariable logistic regression was used to examine the association between each health care dimension and cervical cancer screening in separate models, and then simultaneously in a fully adjusted model.

## Results

A total of 1,236 women participated in the South Africa WHS, and 274 (22.2%) of them had received a pelvic exam with or without a pap test within the last 3 years. Overall among all study participants, the majority of the women had a secondary school education or less, and only about 13% had at least a college degree (Table 1). About 56% of the women resided in an urban area, and almost 70% reported being in good overall health. Almost 70% of the women were currently unemployed for pay, while a slight majority (28%) belonged to the lowest household SES quartile.

**Table 1:** **Socio-Demographic characteristics of screened and unscreened Women, 2003 South Africa World Health Survey**

	**Pelvic/Pap Screening**			
**Variable**	**Total (n = 1,236)**	**Screened (n = 274)**	**Unscreened (n = 918)**	**p-value**
Age (years) Mean (Std. Error)	38.3 (0.6)	36.2 (1.3)	37.3 (0.6)	*0.001*
Education (%)				
Primary school or less	43.4	24.4	47.4	*0.001*
Secondary school	43.9	45.6	44.7	
College or more	12.7	30.0	7.9	
Marital Status (%)				
Never married	48.2	36.3	53.4	*0.001*
Married	35.6	47.8	33.0	
Separated/Divorced	6.6	11.6	5.3	
Widowed	9.6	4.3	8.2	
Region (%)				
Rural	43.8	33.4	47.1	*0.25*
Urban	56.2	66.6	52.9	
Overall Health (%)				
Good	66.8	75.4	65.9	*0.44*
Moderate	23.6	17.1	24.4	
Poor	9.7	7.6	9.7	
Employment (%)				
Government	8.8	17.8	6.5	*0.001*
Non-government	17.4	26.9	15.4	
Self-employed	6.1	5.7	6.4	
Unemployed for Pay	67.7	49.5	71.7	
Household SES (%)				
Q1-Low	27.6	25.7	28.0	*0.07*
Q2	26.5	20.5	28.4	
Q3	25.9	23.0	26.5	
Q4-High	19.9	30.8	17.1	

There were significant differences between screened and unscreened women with respect to education, marital status, employment status, and household SES. Women who had been screened were more likely to have a college degree (30.0% vs. 7.9%, p = 0.001), more likely to be married (47.8% vs. 33.0%, p = 0.001), and were less likely to be unemployed for pay 49.5% vs. 71.7%, p = 0.001). Among screened women, 30.8% belonged to the highest SES quartile compared with only 17.1% of unscreened women (p = 0.07).

Table 2 provides a list of the dimensions as well as the South Africa WHS items used for measurement. Table 3 presents the distribution of the health care access dimensions among participants, and is limited to women who had been to a healthcare facility in the past 12 months and responded to questions regarding the healthcare system. A total of 256 (21%) women ages 18–69 years had been to a health care facility in the past 12 months for themselves or their children, and 25% of these women had received a pelvic exam with or without a pap test within the last 3 years. Overall, less than 5% of women who attended a health care facility in the past year had a form of health insurance. The average number of providers available was 27.5, the majority of providers seen were medical doctors (53.1%), and most of the health care facilities were government-run (67.9%). About 56% of the women traveled for 25 minutes or less to get to the health care facility, and the most common mode of travel was public transport (40.3%). Over 90% of the women were satisfied both with their provider’s skills, and with the availability of medical equipment. Most of the health care providers seen were female (53.8%), and very few participants reported feeling that they were treated worse due to their gender (3.5%), social class (8.1%), or ethnicity (5.4%).

**Table 2:** **Access to health care dimensions in the 2003 South Africa World Health Survey**

**Health Care Access Dimensions**	**Definition**	**WHS Item and Question Number**
Affordability	Ability and willingness to pay for health care provider charges	• Health insurance coverage (Q0600)
		• Household SES (Q0700)*
Availability	Presence of health care providers and type of health care facilities	• Number of health care providers (Q7002)
		• Provider Type: Medical Doctor/Others (i.e. nurses, midwives, traditional healers) (Q7302)
		• Government/ Non-government facility (Q7301)
Accessibility	Geographic distance and convenience of travel to health care center	• Travel time (Q7307)
		• Travel mode (Q7308)
Accommodation	Perception of adequacy of provider’s operation in terms of skills, supplies or drugs	• Provider skills (Q7304)
		• Equipment (Q7305)
Acceptability	Comfort with provider characteristic; Perception of inferior treatment;	• Provider’s gender (Q7303)
		• Perceived worse treatment based on sex, social class, or ethnic group (Q7328-7334)

*Household SES was created as a composite variable based on ownership of permanent household assets.

**Table 3:** **Distribution of health care access dimensions among women who visited a facility in past 12 months, 2003 South Africa World Health Survey**

		**Pelvic/Pap Screening**		
**Variable**	**Total Sample (n = 256)**	**Screened (n = 65)**	**Unscreened (n = 191)**	**p-value**
**Affordability**				
Health Insurance (%)				*0.6*
Yes	4.8	3.8	5.1	
No	95.2	96.2	94.8	
**Availability**				
Number of Health Care providers				
Mean (Std. Error)	27.5 (7)	125 (83)	12.4 (7)	*<0.001*
Facility type (%)				*0.002*
Government	67.9	40.9	77.1	
Non-Government	32.1	59.1	22.8	
Provider type (%)				*0.003*
Medical Doctor	53.1	73.1	45.9	
Nurse/Midwife	41.2	19.6	49.0	
Traditional healer	5.6	7.2	5.0	
**Accessibility**				
Travel time (%)				*0.04*
<=25 minutes	55.6	68.4	50.1	
>25 minutes	44.4	31.5	49.9	
Travel mode (%)				*0.002*
Private	24.7	47.2	16.4	
Public	40.3	34.6	42.4	
Bike/Walk	34.9	18.1	41.3	
**Accommodation**				
Provider Skills adequate (% Yes)	94.0	94.7	93.8	*0.75*
Equipment adequate (% Yes)	91.1	94.4	89.6	*0.20*
**Acceptability**				
Provider Gender (% Female)	53.8	43.4	58.0	*0.19*
Gender (% Worse No vs. Yes)	3.5	3.0	3.3	*0.86*
Social class (% Worse No vs. Yes)	8.1	6.1	8.2	*0.49*
Ethnicity (% Worse No vs. Yes)	5.4	6.1	4.8	*0.69*

About 1 in 4 (25.4%, n = 65) of the women who attended a health care facility in the past year got screened for cervical cancer (Table 3). There were significant differences between screened and unscreened women in relation to the affordability, availability and accessibility dimensions of health care access. In terms of affordability, we observed no differences in health insurance status by screening, although screened women tended to be in the highest quartile of household SES compared to unscreened women (p = 0.07). In terms of availability, screened women had significantly higher number of health care providers available compared with unscreened women (mean 125 vs.12, p = <0.001); they were more likely to visit non-government facilities (59.1% vs. 22.8%, p =0.002); and more likely to have seen a medical doctor (73.1% vs. 45.9%, p = 0.003). In terms of accessibility, screened women were more likely to travel 25 minutes or less to the health care facility (68.4% vs. 50.1%, p = 0.04), and were more likely to have a private mode of transportation (47.2% vs. 16.4%, p = 0.002). There were no differences observed between screened and unscreened women with respect to accommodation or acceptability factors in bivariate analysis.

Multivariable logistic regression analyses were used to investigate the association between each of the five health care access dimensions and cervical cancer screening (Table 4). We examined affordability factors and found no association between household SES and health insurance on cervical cancer screening (Model 1). All three availability factors were associated with screening (Model 2): a higher number of health care providers was positively associated with screening (OR for 1 unit increase in number of providers = 1.01, 95% CI 1.00, 1.01), seeing a nurse/midwife was associated with a lower likelihood of getting screened compared with seeing a medical doctor (OR = 0.33, 95% CI 0.12, 0.86), and visiting a non-government facility was associated with a significant increase in likelihood of getting screened compared with visiting a governmental facility (OR = 2.56, 95% CI: 1.18-5.57). In the accessibility dimension, public transportation (OR = 0.39, 95% CI 0.12, 1.32) or biking/walking (OR = 0.28, 95% CI: 0.09, 0.87) as the primary mode of transportation were negatively associated with screening compared with private transportation (Model 3). We observed no association between accommodation and screening (Model 4). In the acceptability dimension, perception of worse treatment based on personal gender and social class were not associated with screening. Paradoxically, women who reported not being treated worse by their providers based on their ethnicity were less likely to be screened compared to those women who did perceive worse treatment based on ethnicity (OR = 0.26, 95% CI: 0.08, 0.84) (Model 5).

**Table 4:** **Multivariable associations between health care access dimensions and pelvic/pap screening, 2003 South Africa World Health Survey (Odds Ratios and 95% Confidence Intervals)**

	**Age-Adjusted** ^**1**^ **OR (95% CI)**	**Fully-Adjusted** ^**2**^ **OR (95% CI)**
**Affordability**		
**Household SES**		
Q4-High	Ref	Ref
Q3	1.16 (0.3-5.1)	0.79 (0.22-2.83)
Q2	0.34 (0.1-1.0)	0.57 (0.09-3.36)
Q1-Low	0.59 (0.2-1.5)	0.98 (0.21-4.56)
**Health Insurance**		
Yes	Ref	Ref
No	1.87 (0.5-7.2)	0.72 (0.14-3.77)
**Availability**		
**# Health Care providers**	1.01 (1.00-1.01)	1.01 (1.00-1.01)
**Facility type**		
Government	Ref	Ref
Non-Government	2.56 (1.18-5.57)	1.22 (0.48-3.09)
**Provider type**		
Medical Doctor	Ref	Ref
Others (Nurses/midwives)	0.33 (0.12-0.86)	0.13 (0.04-0.42)
**Accessibility**		
**Travel time**		
<=25 minutes	Ref	Ref
>25 minutes	0.59 (0.28-1.26)	1.46 (0.58-3.71)
**Travel mode**		
Private	Ref	Ref
Public	0.39 (0.12-1.32)	1.19 (0.47-2.97)
Bike/Walk	0.17 (0.05-0.57)	0.62 (0.15-2.64)
**Accommodation**		
Provider Skills adequate		
(Yes vs. No)	0.79 (0.19-3.32)	0.40 (0.02-9.65)
Equipment adequate		
(Yes vs. No)	1.99 (0.60-6.63)	1.11 (0.04-20.1)
**Acceptability**		
Provider Gender (Female v. Male)	0.59 (0.23-1.55)	2.81 (1.33-5.92)
Gender (Worse No vs. Yes)	1.19 (0.29-4.92)	2.11 (0.41-11.04)
Social class (Worse No vs. Yes)	2.63 (0.95-7.25)	4.03 (0.30-5.9)
Ethnicity (Worse No vs. Yes)	0.26 (0.08-0.84)	0.17 (0.01-3.82)

^1^Each health care access dimension was assessed in separate models, adjusted for age.

^2^All health care dimension explanatory variables were combined in the fully adjusted model, adjusted for age.

In Model 6, all the health care access dimensions are considered simultaneously in age-adjusted models. Only the availability dimension of health care remained associated with screening. Every unit increase in the number of health care providers available increased the likelihood of screening by 1% (OR = 1.01, 95% CI: 1.00, 1.01). In addition, seeing a nurse/midwife compared to a medical doctor reduced the likelihood of screening by 87% (OR = 0.13, 95% CI: 0.04, 0.42) (Table 4, Model 6).

## Discussion

Using the 2003 South Africa World Health Survey, we examined the association between multiple dimensions of health care access and cervical cancer screening among adult women. We observed that only about 1 out of 5 women included in this study had been screened for cervical cancer in the past 3 years. Among women who had visited a health care facility in the past 12 months, our results suggest that availability, specifically the number of health care providers available as well as the type of provider, was the most significant dimension of access to care associated with screening. Every extra health care provider available in the past year was associated with a 1% increase in likelihood of cervical cancer screening; and a visit to a non-physician health care provider (e.g. nurses, midwives, traditional healers) in the past year was associated with 87% decline in likelihood of cervical cancer screening. These associations remained even after adjusting for other health care access components. We found no significant association in adjusted models between cervical cancer screening and affordability, accessibility, accommodation and acceptability dimensions of access.

Multiple studies have examined screening rates and factors associated with cervical cancer screening across several African countries [10,11,28-36], with reported cervical cancer screening rates as low as 6% in Kenya [36] and 12% in Uganda [35] among adult women. Our observed screening rate of 20% is similar to rates reported in other studies for South Africa [37,38], and only slightly better than other sub-Saharan African countries [39,40]. However, these numbers likely mask large within-country differences in screening rates. For instance, Maree et al. [41] observed that only 4% of women targeted for cervical cancer screening in a poor region of South Africa actually received screening. More recent figures from a separate analysis by Adonis et al. in 2012 revealed a similar picture in South Africa [42]; while prostate cancer screening rates were close to 40% among adult men, the highest cervical cancer screening rate observed was 23% in Gauteng province, compared with 13% in Northern Cape [42].

Several studies have also investigated barriers to cancer screening in general, and cervical cancer screening specifically [28,30,33,35] among African women. The most common barriers to routine screening highlighted in these studies include lack of knowledge about cervical cancer or screening, fear of abnormal results, cost and lack of adequate medical infrastructure and personnel. Our study adds an important component to this area of research by highlighting specific dimensions of health care access that may be important target areas for policies designed to facilitate cancer screening. We found no association between our affordability component and health care screening and no significant results for the accessibility, accommodation, and acceptability components. We did observe that access to a higher number of physicians significantly predicted receipt of cervical cancer screening. Our finding that cost issues (affordability component) and other patient level factors (captured in the acceptability and accommodation components) were less important predictors of screening compared with availability of physicians and physician recommendation has also been observed in prior studies of populations eligible for free cancer screening [43,44].

There were several strengths and limitations of this analysis. First, the study benefited from the standardized protocol used in the World Health Survey, and the stratified clustered sampling technique that ensured representation of participants across South Africa. Secondly, the use of a composite measure of SES contributes to the literature by providing a more robust measure useful in areas where income and education may not fully capture the full extent of an individual/household SES. A limitation of this study is the use of questionnaire data to solicit information, and which may be vulnerable to recall and/or information bias. However, since these questions were part of a larger survey assessing a range of health behaviors, we do not anticipate a high likelihood of socially desirable answers to questions relating to cervical cancer screening. In addition, we were unable to examine results based on if care was being sought for a child or for an adult. It is likely that perceptions about healthcare quality will differ depending on the type of clinic attended (e.g. pediatric or reproductive clinic), or depending on who is receiving care. Finally, the data used for this analysis were collected between 2002 and 2004, and while certain aspects may have changed in the interim, our results provides a deeper understanding of structural issues relating to availability, accessibility, accommodation and acceptability that often take many decades to resolve. Recent studies conducted between 2011 and 2013 indicate that cervical cancer screening rates are still very low in South Africa [37,41,42].

We based our outcome measure on a combined item assessing pelvic examinations with or without a Pap test. Although the questionnaire was designed to assess cervical cancer screening, it is possible that a pelvic examination was done outside the context of cervical cancer screening. However, we observed that of the 274 women who received a pelvic exam, 210 had also reported receiving a Pap test suggesting that the majority of pelvic examinations were being done in the context of cervical cancer screening. We ran a sensitivity analysis on the 210 women who received a Pap test to determine if the regression results would change, yet found similar results. Therefore, we present results based on the analysis of the full set of 274 women assumed to have received a form of cervical cancer screening in South Africa.

Many African countries have very few medical doctors in relation to their population, with some countries well below the WHO minimum of 23 health care workers per 10,000 [45]. These shortages are driven mainly by ‘brain drain’ of health professionals to developed countries, and because existing health care workers also tend to cluster in urban, highly developed areas of the country, leaving the poor, rural areas underserved. An attempt to address this issue has led to ‘task-shifting’, in which professional nurses are tasked with performing the bulk of routine medical care and screening [46,47]. This has had limited success in South Africa, as reflected in the persistently low cervical cancer screening rates, and the significantly lower likelihood of getting screened if women are seeing non-physician providers. This suggests that meeting cervical cancer screening goals and the success of HPV vaccination programs requires significant investments in the health care workforce, improving health care worker density in poor and rural areas, and improved training of the existing workforce.

## Conclusions

Cervical cancer screening rates in South Africa are much lower than the national screening goal or targets set by the WHO. This is likely due to a lack of available healthcare providers, especially medical doctors, which in turn reduces screening access for women eligible for screening.
